# Remote Control System for Battery-Assisted Devices with 16 nW Standby Consumption

**DOI:** 10.3390/s19040975

**Published:** 2019-02-25

**Authors:** Manuel Ferdik, Georg Saxl, Erwin Jesacher, Thomas Ussmueller

**Affiliations:** Microelectronics and Implantable Systems Group, Department of Mechatronics, University of Innsbruck, 6020 Innsbruck, Austria; Georg.Saxl@uibk.ac.at (G.S.); Erwin.Jesacher@student.uibk.ac.at (E.J.); thomas.ussmueller@uibk.ac.at (T.U.)

**Keywords:** energy harvesting, internet of things, low-power electronics, radio frequency, switching circuits, wireless communication

## Abstract

One of the biggest impacts of the vision ‘Internet of Things’ is the massive number of connected devices, where billions of nodes will exchange data, information and commands. While wireless systems offer advantages such as increased flexibility, they also introduce one major challenge: how to power each individual node. In many cases, there is no way around the use of batteries. To minimize the environmental impact, increasing the battery’s longevity is the most important factor. This paper introduces a wireless battery-assisted node that has a drastically reduced energy consumption in the standby mode. The state (on/off) will be changed by harvesting a radiofrequency signal. A latching switch connects or disconnects the load—for example, a microcontroller—and the battery. The switch is connected to a charge pump which converts an AC (alternating current) signal into a usable DC (direct current) control signal. An antenna is mounted to the charge pump via a matching network. An electromagnetic wave is emitted by a remote control switch that switches the system on and off. The used frequency is 868 MHz and therefore in the UHF RFID (ultra high frequency radio frequency identification) band. The measurement results show that the wireless node consumes less than 16nW in the standby mode. The remote controlling is possible from a distance of more than 12m. The presented system can be integrated in further work on a UHF RFID tag. Thus, the existing protocol standard can be used to identify the object to be switched. By custom commands, the switching request can be transmitted from the remote control (UHF RFID reader) to the switching node.

## 1. Introduction

The Internet of Things (IoT) describes a network of billions of devices. Each single node can be a small sensor, a microcontroller (μC) as well as a whole laptop or smartphone. A wired connection of the individual participants in this network would be impractical in many ways. Lack of flexibility, material and installation costs lead to a growing focus on wireless systems. One of the biggest challenges for radio systems is the energy supply. Without the presence of a cable at the node, only the options ‘battery’ and ‘battery-less (or passive)’ are available.

Battery-less systems use energy harvesting to power the passive node and transmit data. Energy sources can be solar radiation, mechanical motion or the electromagnetic wave transmitted from a reader [1]. A promising technology for the latter one is ultra high frequency radio frequency identification (UHF RFID). The lack of battery leads to great ecological as well as economic advantages [2]. However, passive systems are very limited in their functionality. The small amount of energy available leads to a trade-off between reading range and complexity of the task to be performed. Smaller sensors such as temperature sensors [3] can be read out over several meters, but complex microcontrollers cannot be sustainably supplied with energy in that way.

For this reason, wireless nodes typically use a battery as an energy source. In order to minimize both the environmental footprint and the cost (maintenance and material) of battery-based systems, it is essential to maximize battery life. Therefore, nodes should only be enabled when they are really needed and should be in standby for the rest of the time. To achieve this, there are two possible approaches:

1. Remote Control (RC): To switch a device ‘on’ and ‘off’ via the air interface, a remote control can be used which wakes the device up from standby or switches it to standby. An example for this method is the infrared RC in TV’s or DVD players. However, devices in standby must scan the environment for switch-on signals, which leads to a non-negligible power consumption. Although the European Union’s ‘Ecodesign Directive (2009/125/EC)’ has limited the tolerable standby consumption to 0.5W in 2013, it is still too high, considering the sheer number of IoT devices.
2. Target Time: With this option, the node that is in standby mode decides itself when to wake up. Afterwards, it sends a signal to the network to indicate its ‘on’ and then remains in receive mode before returning to standby after a certain time. The internal wake-up logic requires little resources, resulting in a nearly ‘Zero-Watt’ standby mode. However, this method also has disadvantages. The device cannot be woken up by an external device such as a remote control and is therefore limited to fixed time slots. Furthermore, the node is woken up even if it is not necessary, which leads to additional energy consumption. This method is used in LTE-M, which is specified for IoT [4].

The widespread state of the art for remote control systems is based on infrared transmission. As already mentioned, the energy consumption regulated by the EU is 0.5W, which still leads to an extremely high total consumption of standby electricity. A system that reduces standby consumption to 3.9mW has been previously introduced [5]. Boaventura et al. use RF for the remote control system, but concentrate on the RC part and not the standby consumption in the device to be switched [6,7]. Other publications also focus on ‘Zero-Standby’ power switching using RF [8]. The results show a standby current of <10nA at an undefined voltage. This lack of information and the neglect of leakage currents as in [9,10,11] leads to the fact that no final statement can be made about the standby consumption. Approaches to increasing battery life were also presented in [12]. Special attention has been paid to power management and power control with a two factor distance and link factor in order to minimize the transmission power.

However, battery-free systems also have to save energy. Even if the energy is obtained from the environment, this can be a critical factor. Solar energy, for example, is not continuously available and must therefore be buffered by the battery or capacitor [13]. In [14], a predictive power management framework is shown, which combines the optimal working point, deviation aware predictive energy allocation and energy efficient transmission power control. This makes it possible to reduce the system power loss up to 17.49% and improves the transmission energy efficiency up to 23.22% compared with state-of-the-art transmission power control schemes. The development of a reliable energy monitoring and prediction algorithm is also investigated by [15]. Therefore, it is possible to control the power management unit to avoid battery exhaustion even by time-varying solar power. Refs. [16,17] also show a possibility to predict the available energy and use the additional information to adjust the data rate. A hybrid system using solar and electromagnetic energy sources is presented in [18]. In order to minimize energy consumption, monitoring of the radio channel was implemented to adjust the transmission power according to the channel conditions.

The aim of this paper is to combine the advantages of both batteryless and battery-assisted, approaches and additionally to eliminate the disadvantages. The presented 5V battery-assisted wireless node has an standby consumption of ~16nW and can furthermore be woken up by a remote control. The operating frequency is 868 MHz and the maximum allowed transmission power of the RC is 2W. The used switch needs to operate in latching mode to keep its on/off state. Without this feature, the node would only be powered as long as an RC sends a continuous wave (CW) [19]. The presented prototype is implemented on a printed circuit board (PCB). The proposed system can be used for both batteryless and battery-assisted devices. Within this paper, the prototype is designed for latter one. This approach can be seen as additional power saving technique for the above mentioned related research. The frequency was specifically selected from the UHF RFID band to integrate the switch in future work into a complete UHF RFID Tag. Thus, the EPCglobal Gen2 V2 specification [20] can be used for the identification of the object to be switched. The switching request can be implemented by a custom commands.

## 2. Implementation

**Figure 1:**
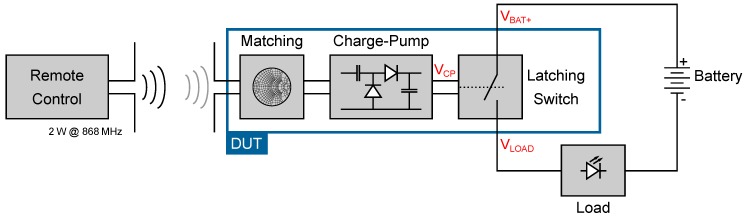
Schematic representation of the proposed remote control system.

As shown in Figure 1, the presented system consists of several components. A remote control transmits a 2W CW at 868 MHz. The wireless node receives the electromagnetic wave through an antenna which is connected to a charge pump over a matching network. The charge pump converts the AC (alternating current) signal of the antenna into the DC (direct current) voltage $V_{CP}$, which is used to control a switch. The switch acts as a latching switch and disconnects or connects a battery to the load. In standby mode, the consumption of the switch should be as low as possible to maximize the persistence of the battery. Each component of Figure 1 will be discussed in the following section.

### 2.1. Latching Switch

**Figure 2:**
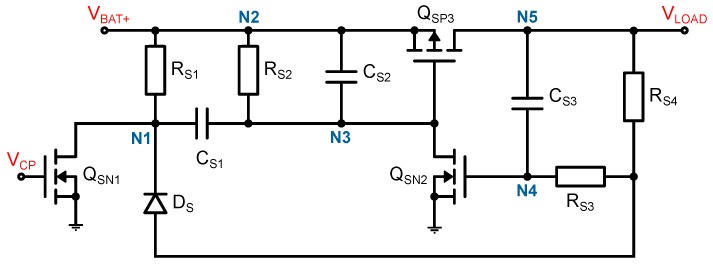
Schematic representation of the latching switch. The transistor $Q_{SP3}$ disconnects or connects the battery to the load. The switching command therefor is applied to the gate of $Q_{SN1}$.

The latching switch (LS) is the core component of the presented system as the standby consumption depends exclusively on it. The LS should connect a 5V battery to the load. The schematic of the switch is shown in Figure 2. The charge pump (CP) provides a 1V pulse which can be used as input and switching command. Only a short pulse will turn the system on, while the switching-off process takes 1.4s. This is intended to prevent accidental turning off, which can cause fatal errors in certain loads (e.g., μC). Both time constants can be adjusted with the resistors and capacitors (switch-on with $C_{S1}$ and $R_{S1}$ and switch-off with $C_{S3}$ and $R_{S3}$). The values in Table 1 were used for the proposed prototype. When selecting the transistors, care was taken to ensure that all MOSFETs (metal-oxide-semiconductor field-effect transistor) have a low leakage current between drain and source. This leakage current is the main energy consumer in the standby mode and therefore extremely important. In addition, a minimal threshold voltage for $Q_{SN1}$ was chosen in order to ensure a quick reaction of the LS. The switch is resistant to hysteresis, which means that the target state is kept also for longer switching pulses and does not change again after a certain time. The working principle of the LS in Figure 2 will be described in the following [21].

- Initial State: When no AC signal is applied to the CP, $V_{CP}$ is floating and therefore no gate-source-voltage is applied to $Q_{SN1}$. When the battery gets connected ($V_{BAT+}=5\;V$), the capacitor $C_{S2}$ shorts node N3 with $V_{BAT+}$ at first and thus determines the standard starting state. This prevents a floating node, which would inadvertently switch on $Q_{SP3}$. The potential of the nodes N1, N2 and N3 is $V_{BAT+}$. Therefore, the PMOS (P-type metal-oxide-semiconductor) transistor $Q_{SP3}$ blocks and the potential of node N5 will be pulled to ground (GND) via the load. Thus, N4 is also on the same potential and the gate of $Q_{SP3}$ will not be connected to GND over $Q_{SN2}$. The off state will be kept and $V_{LOAD}$ is on the same potential as GND.
- Switching ON: When a voltage $V_{CP}>V_{TH}$ is applied to $Q_{SN1}$, it pulls the node N1 to GND. In the first moment, the capacitor $C_{S1}$ acts as a short and thus N3 will also become GND which allows $Q_{SP3}$ to connect. The $V_{BAT+}$ potential of N5 is applied to N4 via $C_{S3}$ which causes $Q_{SN2}$ to connect through and pull the gate of $Q_{SP3}$ permanently to GND. Even if $V_{CP}=0$ again, the on state will be kept and $V_{LOAD}=V_{BAT+}$.
- Switching OFF: When a voltage $V_{CP}>V_{TH}$ is applied to $Q_{SN1}$, it pulls the node N1 to GND. The capacitor $C_{S3}$ will be charged via the diode $D_{S}$ and pulls N4 towards GND. When the potential at N4 is falling below the threshold voltage of $Q_{SN2}$, it stops connecting through and the gate of $Q_{SP3}$ will be high again. The battery is disconnected from the load and the off state will be kept.

**Figure 3:**
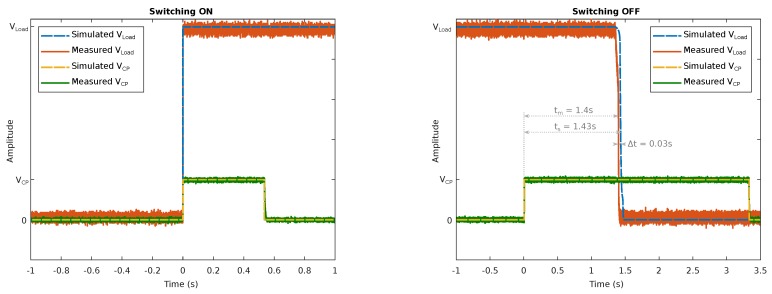
Simulation and measurement results of the switching process. When a voltage $V_{CP}$ is applied, the circuit reacts and the battery is connected or disconnected to the load.

The results of the LS simulation are shown in Figure 3. Both the switch-on and the switch-off process are plotted. The circuit works as desired. Even a short pulse at the $V_{CP}$ switches the system on. Switching off in turn requires a pulse of at least 1.4 s. If a pulse longer than 1.4 s is applied, the system will not switch on again.

The advantage of the presented topology over other topologies as shown in [22] is the beneficial placement of the ‘input transistor’ $Q_{SN1}$. In our case, the source is permanently grounded. Thus, $V_{GS}$ is equal to $V_{CP}$. In [22], the source potential is time and state dependent and leads to $V_{GS}$ being unequal to $V_{CP}$. This means that a different $V_{CP}$ is required at different times, which makes implementation more difficult.

### 2.2. Charge Pump

The charge pump provides the voltage required to operate the LS. It converts the AC signal of the antenna into a DC voltage which is suitable for the following circuit. The chosen input transistor $Q_{SN1}$ of the switch has a threshold voltage of more than 0.4V. In order to guarantee a certain safety factor, the CP is dimensioned in such a way that it supplies 1V at a 10m distance. Since the $Q_{SN1}$ is a MOSFET, ideally no currents flow into the gate. Only the input capacitance has to be charged to switch the transistor.

The CP is fed by the antenna with an 868 MHz CW signal. A three-stage cascaded Greinacher circuit is chosen as the topology. The number of stages ensures that even an input signal as low as 0.22V is sufficient to meet the requirements of the LS. Schottky diodes (see Table 1) were used which only require a low forward voltage of 150 mV and at the same time are suitable for the chosen frequency.

### 2.3. Matching Network and Antenna

**Figure 4:**
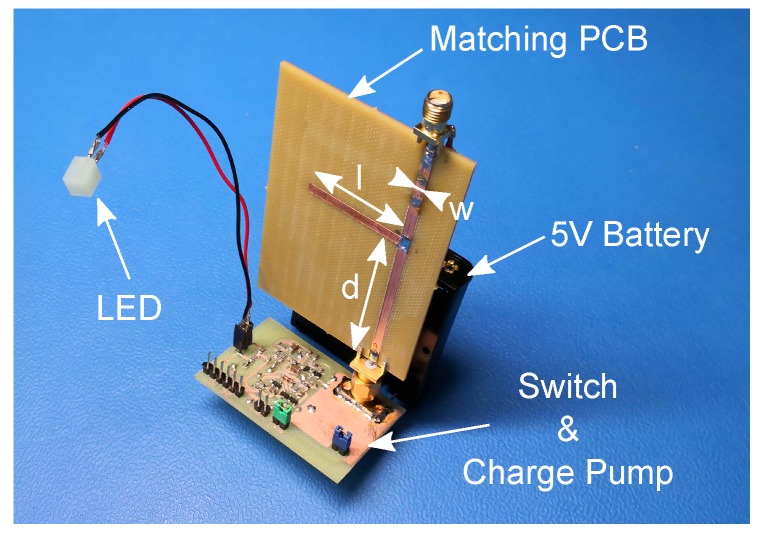
PCB (printed circuit board) prototype of the proposed system. An LED is used as the load, driven by a 5V battery. The microstrip stub tuner (matching network) is mounted to the CP (charge pump) via a SMA connector.

The matching network is responsible for matching the impedance of the node (including CP, LS, load and battery) to the impedance of the antenna. Due to the high frequency, a matching based on a microstrip stub tuner is used, which consists of a stub and a transmission line to match input and output impedance. In the present paper, two different measurements were performed. In order to use the same matching network for both setups, an antenna with the same impedance ($Z_{0}=50\;\Omega$) as the signal generator was chosen. The matching procedure can be subdivided as follows:

1. **Input Power for Matching**: Since the input impedance of the circuit depends on the input power, this must be determined in the first step. A common approach is presented in [23]. First, the unmatched circuit is connected to a digital signal generator (DSG) via cable (Sucoflex 104, 0.5m) to get the minimum needed power $P_{unmatch}$ to control the actuator. This power is used as output power at the vector network analyzer (VNA) to determine the corresponding $S_{11}$. The calculated sensitivity of the actuator is then derived from [23]
  (1) $$P_{sens,calc}=P_{unmatch}\;\cdot \;\left(1-|S_{11}{|}^{2}\right)=-10.97\;\mathrm{dBm}.$$
2. **Actuator Impedance Determination**: The input impedance $Z_{A}$ of the actuator circuit for $P_{sens,calc}=-10.97\;\mathrm{dBm}$ has been determined with a network analyzer and results in:
  (2) $$Z_{A}=R_{A}+jX_{A}=142.9\;\Omega -j115.9\;\Omega .$$
3. **Matching Network Calculation**: Due to the high frequency, a single microstrip stub tuner is used for the matching of the actuator impedance $Z_{A}$ with $Z_{0}=50\;\Omega$. The geometry of the matching can be seen in Figure 4. The two dimensioning parameters *d* and *l* can be calculated according to [24] and lead to:
  $$d=0.289\;\lambda_{eff},\;\;l=0.167\;\lambda_{eff}.$$
  The effective wavelength $\lambda_{eff}$ depends on the PCB material and the microstrip width *w*. For the calculations, the ‘PCB Toolkit’ from ‘Saturn PCB Design’ has been used. To achieve a 50 Ω wave impedance on our ‘Panasonic R-1566 FR4’ [25] laminate for 868 MHz, the width *w* needs to be 2.85mm. Therefore, the effective wavelength $\lambda_{eff}=184.1\;\mathrm{mm}$ and this results in the final parameters:
  (3) d=53.29mm,l=30.87mm,w=2.85mm.
4. **Simulation**: With the matching network parameters (3) and the actuator impedance (2), a simulation has been performed with the software ‘Qucs 2.5.7’. The results show that the matching works and the impedance is transformed to $Z_{A,match}=53.2\;\Omega -j1.8\;\Omega$.
5. **Fabrication**: The circuit was fabricated on the selected laminate and SMA connectors were mounted on the PCB (see Figure 4).
6. **Verification**: For verification, the actuator and the matching network were connected to the network analyzer. The results show there is a good agreement between calculation, simulation and measurement. An impedance of 52.0Ω−j1.1Ω has been measured, which indicates a proper matching.

### 2.4. Device under Test

All circuits of the proposed prototype, including LS, CP and matching network, are fabricated on ‘Panasonic R-1566 FR4 Laminate’ [25]. The device under test (DUT) for all performed measurements is shown in Figure 4. A LED was used as load, driven by a 5V battery.

## 3. Measurement Setup

**Figure 5:**
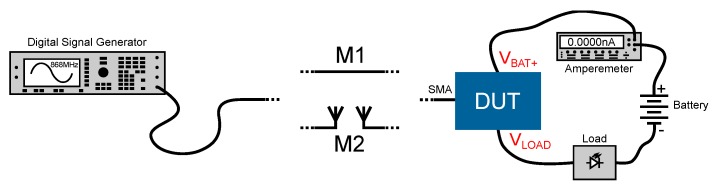
Schematic representation of the measurement setups. A signal generator generates an 868 MHz CW signal for the DUT. In the first setup (M1), the signal is transmitted via cable, in the second one (M2) via antennas.

**Figure 6:**
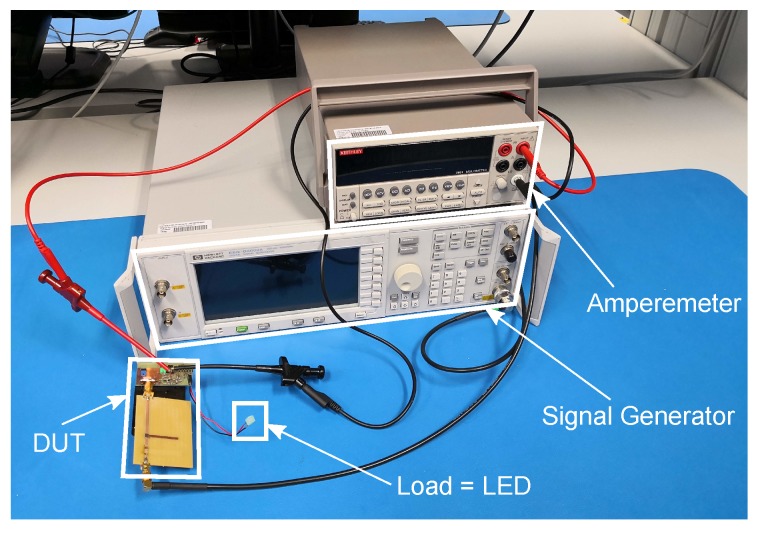
Measurement setup M1. A simple LED with series resistor was used as load.

Two different measurements were performed to characterize the presented system. The measurement setup is shown in Figure 5. For measurement M1, the DUT was connected to the DSG ‘HP ESG - D4000A’ via cable. The DSG is used to generate the 868 MHz CW signal. To measure the standby consumption, an amperemeter was connected between DUT and battery. Figure 6 shows a photo of measurement setup M1.

The second measurement setup also considers the air interface. The matching network of the DUT is connected to a ‘Siemens Simatic RF640A’ antenna and a ‘Siemens Simatic RF660A’ antenna is mounted on the DSG. The remaining structure is identical to M1.

## 4. Results

### 4.1. Wired (M1)

After performing the measurement M1, the basic functionality of the system can be ensured. The load can be switched on and off by generating a signal from the DSG and sending it to the DUT. The measurements showed a sensitivity of the actuator of $P_{sens,meas}=-10.94\;\mathrm{dBm}$. Therefore, a 868 MHz signal with $P>P_{sens,meas}$ is sufficient for switching the device on or off. The voltage over time for switching the LS on and off is shown in Figure 3. This shows a good agreement between measurement and simulation. During the switch-off process, the delay to the switching pulse is 30ms (≈2.1%) longer than during the simulation. Since this time reacts sensitively to the dimensioning of the components, the deviation can be attributed to component tolerances in the LS. The most important characteristic of the system—the standby consumption—was measured using a high-precision amperemeter. When switched off, the 5V battery is strained with 3.2nA. This results in a total standby power consumption of 16nW.

### 4.2. Wireless (M2)

**Figure 7:**
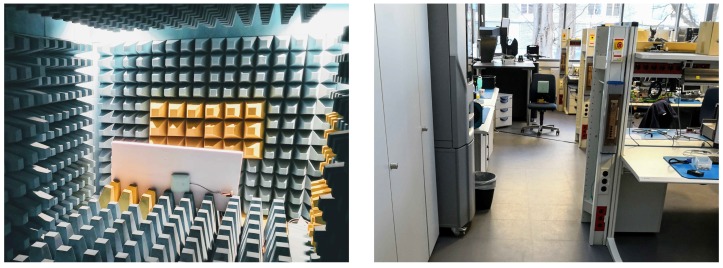
Measurement setup M2. On the left side, the actuator has been placed in the anechoic chamber and on the right side for multipath propagation in a laboratory.

The second measurement setup M2 also takes the air interface between DSG and DUT into account. This setup was used to perform two different measurements. First, the relationship between the distance and the received power was investigated in an anechoic chamber (see Figure 7—left). In the second step, the maximum range of the system was determined in a realistic environment akin to the situation in a consumers living room, with plenty of possibilities for multipath propagation (see Figure 7—right).

#### 4.2.1. Anechoic Chamber Measurement

The aim of the first measurement is to investigate the correlation between the operating range of the system and the received power at the actuator. The measurements were performed in an anechoic chamber to avoid possible multipath reception and interferences from other radio sources. Limited by the dimensions of the chamber, the distance between actuator and DSG antenna was fixed at 2.34m. After the sensitivity of the actuator is already known (see Section 4.1), the Friis equation [26] can be reshaped and the minimum transmission power is calculated:

(4) $$\begin{array}{ccc} P_{RC,calc}\left(R=2.34\;m\right) & = & P_{sens,meas}-G_{A}-G_{RC}-20\;\cdot \;\log_{10}\left(\frac{\lambda }{4\pi R}\right)\\ & = & -10.94\;\mathrm{dBm}-7\;\mathrm{dBi}-4\;\mathrm{dBi}-20\;\cdot \;\log_{10}\left(\frac{0.3454\;m}{4\pi \;\cdot \;2.34\;m}\right)\\ & = & 16.66\;\mathrm{dBm}. \end{array}$$

To measure the minimum transmission power in the anechoic chamber, the output power of the DSG was incrementally increased. The results showed that the measured $P_{RC,meas}\left(R=2.34\;m\right)=16.63\;\mathrm{dBm}$ matches well with the calculated power. This corresponds to an relative error of $\varepsilon_{r}=0.2\%$ with respect to the $P_{RC,calc}$.

The same procedure was also performed for the distance R=1.68m. According to Friis, the minimum transmission power for this distance is $P_{RC,calc}\left(R=1.68\;m\right)=13.78\;\mathrm{dBm}$. The measurement in the anechoic chamber results in $P_{RC,meas}\left(R=1.68\;m\right)=13.7\;\mathrm{dBm}$. Again, there is good agreement with a relative error of $\varepsilon_{r}=0.6\%$.

#### 4.2.2. Multipath Environment Measurement

The second measurement with Setup M2 is used to determine the maximum operating range within multipath environmental conditions. The maximum allowed transmission power in the 865–868 MHz frequency band in the EU is 2W = 33dBm ERP (effective radiated power) [27]. The output power of the DSG can be calculated as follows:

33dBmmax. allowed ERP+2.15dBiisotropic gain of a half-wave dipole=35.15dBm  max. allowed EIRP (effective isotropic radiated power)−7dBiRF660A antenna gain+1dBcable attenuation=29.15dBmDSG output power.

The measurements were performed in a common laboratory. Due to the limited dimensions of the lab, the transmitter and the wireless node only can be separated by a maximum distance of $R_{max,meas}=12\;m$. At this distance, the switching process could still be carried out. The standby consumption of 16nW has not changed. According to Friis, the maximum operating range of $R_{max,calc}=8.78\;m$. The difference between measurement and calculation is mainly attributable to the multipath propagation since a good agreement under ideal conditions has already been shown in in the anechoic chamber.

## 5. Conclusions

This paper shows a way to switch battery-assisted wireless nodes on and off in a network by an external remote control using UHF RFID. In order to keep the state, a latching switch topology has been proposed which can be controlled by a single MOSFET transistor. The signal for this transistor is provided by a charge pump, which rectifies an AC Signal and boosts it to a certain DC voltage. The AC signal will be generated by a remote control and transmitted over the air. The wireless node receives the signal over an antenna and forwards it to the charge pump via a matching network.

When turned off (standby mode), the node consumes less than 16nW. When the remote control applies a signal to the node, it switches on and the load will be connected to the battery. To switch it off again, the remote control must transmit a signal again. By selecting the appropriate switch topology, oscillation between on and off is avoided. The maximum operating range could not be determined due to the limited dimensions of the measurement setup. However, the functionality has been shown up to a distance of 12m.

In future work, the presented system can be integrated into a whole UHF RFID tag. This tag can be viewed as an RFID actuator that can switch a subsequent circuit on or off. The switching request can be transmitted from the remote control via a custom command. By integrating the new functionality into an UHF RFID Tag, the standard protocol can also be used to identify the object to be switched. This allows for controlling several devices selectively.

The proposed system can be used in a wide variety of applications. In the present version, nodes that require a 5V DC supply can be switched. These could be, for example, all devices that can also be supplied via USB like microcontrollers. Furthermore, the node can be used to control a subsequent AC circuit. This would enable the control of consumer electronics devices such as TVs. In this area, standby mode has been a problem for some time. The presented system can also be used to wake up batteryless nodes from an almost ‘Zero-Standby’ mode. In particular, nodes harvesting solar radiation have to buffer it to due the time-varying solar power. Therefore, energy-saving techniques are also important.

Thus, the presented system is an ideal candidate to reduce the energy consumption in the Internet of Things and to minimize the impact on the environment. Both battery-assisted and batteryless wireless nodes can be awakened from an almost ‘Zero-Standby’. The shown approach can be used in combination with other state-of-the-art energy saving measures. Especially for nodes that only need to be active a few times a day, energy consumption can be drastically reduced. 

## Figures and Tables

**Table 1:** Used components for the latching switch and charge pump.

Component	Type	Component	Type
$R_{S1}$	10kΩ	$Q_{SN1}$	BSH105
$R_{S2}$,$R_{S4}$	100kΩ	$Q_{SN2}$	BSS138
$R_{S3}$	300kΩ	$Q_{SP3}$	BSS84
$C_{S1}$	1 μF	$C_{CP1}$	10nF
$C_{S2}$	100nF	$C_{CP2}$	100nF
$C_{S3}$	2.2 μF	$D_{CP1}$, $D_{CP2}$	HSMS285
$D_{S}$	1N4148	$D_{CPZ}$	Zener 3.6V
